# POTEC (Platelet Count, Oxygen Saturation, Time of CPR, Elective Surgery, and Initial ETCO_2_) Score for Predicting 24-h Survival After Perioperative Cardiopulmonary Resuscitation: Development and Validation

**DOI:** 10.3390/jcm14196915

**Published:** 2025-09-29

**Authors:** Soontarin Chungsaengsatitayaporn, Tanyong Pipanmekaporn, Jiraporn Khorana, Prangmalee Leurcharusmee, Visith Siriphuwanun, Settapong Boonsri

**Affiliations:** 1 Department of Anesthesiology, Faculty of Medicine, Chiang Mai University, Chiang Mai 50200, Thailand; khunyimkha@gmail.com (S.C.); tanyong24@gmail.com (T.P.); prangmalee.l@cmu.ac.th (P.L.); visith_cmu@yahoo.com (V.S.); 2 Department of Biomedical Informatics and Clinical Epidemiology, Faculty of Medicine, Chiang Mai University, Chiang Mai 50200, Thailand; jiraporn.kho@cmu.ac.th; 3 Division of Pediatric Surgery, Department of Surgery, Faculty of Medicine, Chiang Mai University, Chiang Mai 50200, Thailand; 4 Clinical Surgical Research Center, Department of Surgery, Faculty of Medicine, Chiang Mai University, Chiang Mai 50200, Thailand

**Keywords:** predictive score, perioperative cardiac arrest, survival, anesthesia, POTEC score

## Abstract

**Background**: Perioperative Cardiac Arrest (POCA) is a rare but catastrophic event with persistently low survival rates. Existing prediction models often fail to capture the perioperative context or predict short-term outcomes. This study aimed to develop and internally validate the POTEC (Platelet count, Oxygen saturation, Time of cardiopulmonary resuscitation (CPR), Elective surgery, and initial end-tidal carbon dioxide (ETCO_2_) Score, a simple clinical tool for predicting 24-h survival following perioperative CPR. **Methods**: We conducted a retrospective cohort study of adult patients (≥18 years) who experienced POCA during or within two hours after non-cardiac surgery under anesthesia at a tertiary university hospital between 2010 and 2023. Multivariable logistic regression was used to identify independent predictors of 24-h survival. The final model’s coefficients were used to construct the POTEC Score, which was internally validated using bootstrapping (1000 replications). **Results**: Of 321 eligible patients, 65 (20.25%) survived at 24-h. Five variables were independently associated with 24-h survival and included in the POTEC score: preoperative platelet count 100 × 10^9^/L, preoperative oxygen saturation of ≥90% on room air upon arrival in the operating room, CPR duration ≤30 min, elective surgery, and initial end-tidal CO_2_ between 35 and 45 mmHg. The score demonstrated good discrimination (AuROC = 0.788, 95% CI: 0.73–0.85) and calibration (Hosmer–Lemeshow *p* = 0.535). A score of 4 points or higher was associated with significantly higher odds of 24-h survival (adjusted OR = 2.78, 95% CI: 2.05–3.79). Model optimism was minimal (0.009) after bootstrapping. **Conclusions**: The POTEC Score is a clinically practical tool for early risk stratification in patients undergoing perioperative CPR. Its integration into perioperative workflows may aid in timely decision-making and resource prioritization during critical postoperative care.

## 1. Introduction

Perioperative cardiac arrest (POCA) is a rare but devastating complication associated with anesthesia and surgery. Although advancements in intraoperative monitoring and the presence of trained anesthetic and surgical teams have improved perioperative safety, outcomes following perioperative cardiopulmonary resuscitation (CPR) remain poor and difficult to predict [1,2]. During the past ten years, the incidence of POCA has been reported to range from 4.3 to 9 per 10,000 anesthetic procedures [3,4,5], and 24-h survival rates remain under 40% in high-income countries [1,6,7] and 10% in Thailand [8]. These sobering statistics underscore the urgent need for perioperative-specific tools that facilitate rapid risk stratification and inform critical decision-making during and after resuscitation [1,3,9].

Several biomarkers and early warning scores have been explored for predicting outcomes following cardiac arrest. Biomarkers such as high-sensitivity troponin and serum lactate have demonstrated prognostic value; however, their clinical utility is limited by cost, delayed processing times, and a lack of bedside availability [10,11,12,13]. Early warning systems, including the cardiac arrest risk triage (CART) score [14] and the modified early warning score (MEWS) [15,16], were developed to identify patients at risk of cardiac arrest in ward-based settings. However, respiratory rate and level of consciousness may be less applicable in the perioperative environment. Furthermore, the causes of POCA often involve a complex interplay of surgical events, anesthesia-related complications, and preoperative patient factors, including airway compromise, hemorrhage, cardiovascular collapse, and metabolic disturbances [3,4,7,17,18], which existing models do not fully address.

To date, no clinical prediction model has been specifically developed and validated to predict short-term survival following POCA. Existing tools are limited in their applicability to intraoperative and early postoperative scenarios. Therefore, there is a critical need for a simple, reliable, and perioperative-specific prognostic score that can be applied immediately after the return of spontaneous circulation (ROSC) or early in the post-arrest phase. Such a model could assist in guiding triage, resource allocation, and postoperative care planning. This study aimed to develop and internally validate a novel clinical prediction model, referred to as the POTEC (Platelet count, Oxygen saturation, Time of CPR, Elective surgery, and initial ETCO_2_) Score. The goal of the POTEC Score is to estimate 24-h survival in adult patients who experience POCA during non-cardiac surgery.

## 2. Materials and Methods

### 2.1. Study Design

This retrospective cohort study was conducted by the Transparent Reporting of a Multivariable Prediction Model for Individual Prognosis or Diagnosis (TRIPOD) guidelines for developing and reporting predictive models [19]. Ethical approval was granted by the Ethics Committee of the Faculty of Medicine, Chiang Mai University (Study Code: ANE:2565-08823), with a waiver of informed consent due to the retrospective nature of the study.

### 2.2. Participants

Our study included adult patients aged 18 years or older who experienced POCA during non-cardiac surgery under general anesthesia (GA), regional anesthesia (RA), or monitored anesthesia care (MAC) at Chiang Mai University Hospital between January 2010 and December 2023. Cardiac arrest was defined as the absence of cardiac rhythm requiring CPR, either by closed chest compressions or, in rare cases, open cardiac massage [20,21]. POCA was identified when cardiac arrest occurred intraoperatively or within two hours postoperatively and was confirmed by the attending anesthesiologist or surgical team, ensuring the thoroughness and reliability of our findings. Exclusion criteria included patients who had undergone liver and/or kidney transplantation, were pregnant, or had incomplete or inaccessible medical records. These exclusions were applied because such cases involve distinct physiological conditions that would limit the generalizability of the model to the broader perioperative population.

### 2.3. Outcome

The primary outcome was 24-h survival status following perioperative CPR. This was defined as a binary classification: survivor (alive at 24 h) or non-survivor (deceased within 24 h). This outcome served as the dependent variable in the development of the predictive model estimating short-term survival after perioperative cardiac arrest.

### 2.4. Predictors

Candidate predictors were selected based on clinical relevance, pathophysiological rationale, and evidence from prior studies related to perioperative cardiac arrest and resuscitation outcomes. These variables were categorized into five domains: (1) baseline patient characteristics, (2) preoperative laboratory findings, (3) surgical characteristics, (4) intraoperative physiological monitoring, and (5) resuscitation-related parameters.

Baseline patient characteristics included age, sex, American Society of Anesthesiologists (ASA) physical status, and comorbidities. Preoperative hemodynamic stability was defined as a mean arterial pressure (MAP) ≥ 65 mmHg, heart rate between 50 and 100 bpm, absence of vasopressor use, and no clinical signs of peripheral hypoperfusion [20]. Spontaneous breathing before surgery, defined as the absence of mechanical ventilatory support [21], was also recorded.

Preoperative laboratory variables included hemoglobin level, platelet count, neutrophil-to-lymphocyte ratio (NLR), serum albumin, creatinine, sodium, potassium, blood glucose, and findings from the most recent preoperative 12-lead electrocardiogram (ECG). The most recent lab results available before surgery were used for analysis.

Surgical characteristics data included the urgency of the procedure (elective or emergency), anatomical site (e.g., intra-abdominal, major vascular, intrathoracic, intracranial, or major orthopedic), patient positioning (e.g., supine, lateral, prone), and trauma status. Cases were categorized as trauma or non-trauma, with the latter defined as operations unrelated to acute external injuries such as accidents or assault [7].

The data collection process for intraoperative physiological monitoring was meticulous. Data were collected upon the patient’s arrival in the operating room, before the onset of cardiac arrest. These included initial heart rate, systolic and diastolic blood pressure, MAP, peripheral oxygen saturation (SpO_2_) on room air, and the first documented end-tidal carbon dioxide (ETCO_2_) level following endotracheal intubation. For patients already intubated before operating room admission, the earliest intraoperative ETCO_2_ value was used. Resuscitation-related parameters included the timing of cardiac arrest, a crucial factor in predicting outcomes. Cardiac arrests that occur during working hours [08:00–16:00] may have better outcomes due to the availability of more experienced staff and resources. The initial cardiac rhythm (shockable vs. non-shockable), and total CPR duration, defined as the time from arrest onset to ROSC [22]. In patients with multiple perioperative cardiac arrests, only the first event during the index surgery was analyzed.

### 2.5. Sample Size

The sample size was calculated using the pmsampsize command in Stata version 16.0, following the method described by Matsuyama et al. [23]. Based on our previous study [24], we considered 23 significant parameters, targeting an area under the receiver operating characteristic curve (AuROC) of 0.80 with an expected survival prevalence of 20.7%. The minimum required sample size was estimated to be 248 patients. Given that our retrospective dataset included more than the required number of eligible cases, we made the decision to include the entire population from the defined study period. This was done to ensure the statistical power and precision of the model, demonstrating the thoroughness and reliability of our research.

### 2.6. Missing Data

A complete case analysis was used in the multivariable modeling to ensure data consistency across predictors. Two physiological variables, SpO_2_ and ETCO_2_, measured upon arrival in the operating room were missing in 14 patients (4.36%). These two variables were retained as candidate predictors due to their established prognostic value. Only cases with complete data for these two variables were included in the final model. Therefore, listwise deletion was applied, resulting in a final predictive model based on 303 patients.

### 2.7. Statistical Analysis

Data analysis was conducted using STATA software, version 16.1. (StataCorp LP, College Station, TX, USA). Descriptive statistics were used to summarize baseline characteristics. Continuous variables were assessed for normality using the Shapiro–Wilk test and were reported as mean ± standard deviation (SD) or median with interquartile range (IQR), as appropriate. Categorical variables were presented as frequencies and percentages. Differences between survivors and non-survivors were evaluated using the most appropriate statistical tests: Fisher’s exact test for categorical variables and Student’s *t*-test or Mann–Whitney U test for continuous variables, depending on the data distribution. A *p*-value of less than 0.05 was considered statistically significant.

### 2.8. Model Development

Univariable logistic regression was performed to identify potential predictors of 24-h survival following perioperative CPR. Variables with a *p*-value < 0.10 were included in a multivariable logistic regression model using backward stepwise elimination. Adjusted odds ratios (ORs) with 95% confidence intervals (CIs) were reported, and variables with a *p*-value < 0.05 were included in the final model.

### 2.9. Model Performance and Internal Validation

Discriminative performance of the model was assessed using the AuROC. The level of accuracy is considered acceptable to good in clinical prediction model performance, as an AuROC between 0.7 and 0.8 indicates moderate to good discrimination. While calibration was evaluated using the Hosmer–Lemeshow goodness-of-fit test and a calibration plot comparing predicted and observed outcomes. The diagonal dashed line represents perfect calibration, where predicted probabilities perfectly match observed outcomes. The solid gray line (Lowess-smoothed curve) closely follows the reference line, suggesting that the model’s predictions are well-calibrated across the range of predicted risks. Each dot represents a calibration group, with observed probabilities plotted against mean predicted probabilities. Sensitivity, specificity, positive predictive value (PPV), and positive likelihood ratios were calculated for different modeling score cut-off points. Internal validation was performed using bootstrapping with 1000 replications to assess the model’s robustness and estimate optimism in predictive performance. The model optimism was estimated and reported.

### 2.10. Model Presentation

To create a clinically applicable scoring system, regression coefficients (β) from the final multivariable logistic regression model were transformed into integer-based point values. Each predictor was assigned a score proportional to its β coefficient, rounded to the nearest whole number. The total score was then calculated by summing the individual component points. Further details of the scoring system and risk stratification are presented in the Results section.

## 3. Results

**Figure 1:**
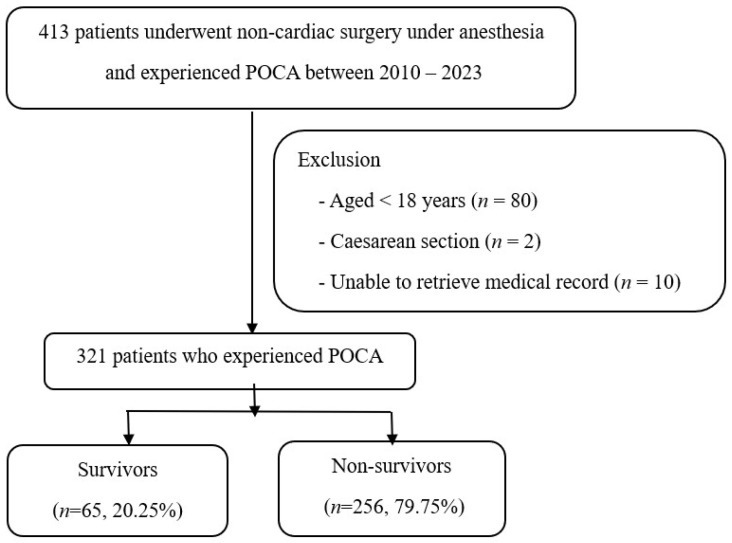
Flowchart of the Studies Included in the Review.

A total of 413 adult patients who experienced POCA following non-cardiac surgery under GA, RA, or MAC between January 2010 and December 2023 were initially identified. This study specifically focuses on adult POCA patients, making it directly relevant to your field of interest. After excluding patients under 18 years of age (*n* = 80), cesarean section cases (*n* = 2), and cases with inaccessible or missing clinical data (*n* = 10), a final cohort of 321 patients was included in the analysis. Among these, 65 patients (20.25%) the survivor group at 24 h following CPR, while 256 (79.75%) were in the non-survivor group. This is shown in Figure 1.

Table 1 presents the clinical characteristics of patients who survived and died within 24h after POCA, along with the univariable logistic regression analyses. Several preoperative clinical variables demonstrated statistically significant associations with 24-h survival following POCA. Patients aged ≥ 65 years had significantly higher odds of surviving compared to those aged < 65 years (OR = 2.58, 95% CI: 1.39–4.79, *p* = 0.003), with moderate discriminative ability (AuROC = 0.602). Female patients exhibited better survival outcomes than males (OR = 2.23, 95% CI: 1.28–3.87, *p* = 0.005, AuROC = 0.596). Patients with an ASA physical status of 1–2 had a substantially greater chance of survival compared to those with an ASA ≥ 3 (OR = 14.96, 95% CI: 5.84–36.92, *p* < 0.001, AuROC = 0.633). Elective surgery was also associated with improved survival, as patients undergoing non-emergency procedures had higher odds of survival (OR = 4.59, 95% CI: 2.17–9.71, *p* < 0.001, AuROC = 0.589).

Preoperative physiological status further influenced survival outcomes. Patients who were breathing spontaneously before surgery had significantly improved odds of survival compared to those requiring ventilatory support (OR = 2.05, 95% CI: 1.14–3.69, *p* = 0.017, AuROC = 0.572). Additionally, patients who exhibited no clinical signs of shock before the arrest event were more likely to survive (OR = 3.33, 95% CI: 1.69–6.52, *p* < 0.001, AuROC = 0.623). Preoperative laboratory variables, including hemoglobin ≥8 g/dL and platelet count ≥ 100 × 10^9^/L, measured from the most recent blood samples before surgery, were significantly associated with increased 24-h survival (OR = 4.73 and 3.97, respectively; both *p* < 0.001). These laboratory values were typically obtained immediately before surgery. However, in trauma or emergency cases, timely collection was often not feasible, leading to 14 cases with missing data. Intraoperative parameters and vital signs recorded upon arrival in the operating room were also predictive of survival. Upon arrival in the operating room, initial vital signs were recorded to establish baseline conditions before any cardiac events. Such as SpO_2_ ≥ 90% (OR = 3.68, *p* < 0.01, AuROC = 0.624) and normal ETCO_2_ 35–45 mmHg, which was measured immediately following successful endotracheal intubation, were significantly associated with better outcomes (OR = 2.79, *p* = 0.004, AuROC = 0.571) were significantly associated with improved outcomes. Finally, a CPR duration (≤30 min) demonstrated the strongest association with 24-h survival (OR = 6.44, 95% CI: 2.26–18.34, AuROC = 0.618) (as shown in Table 1).

All variables with *p*-values less than 0.10 in the univariable analysis were considered for entry into a multivariable logistic regression model. Using backward stepwise selection, five variables remained statistically significant and were independently associated with 24-h survival: platelet count ≥ 100 × 10^9^/L, SpO_2_ ≥ 90% upon operating room arrival, CPR duration ≤ 30 min, elective surgery, and ETCO_2_ level 35–45 mmHg after intubation, were found to be crucial in predicting patient survival. These five predictors formed the basis of a clinical risk score. Regression coefficients were scaled and rounded to assign point values to each predictor, resulting in a cumulative score ranging from 0 to 7. Table 2 summarizes the adjusted odds ratios, regression coefficients, and assigned points.

The scoring system, referred to as the POTEC Score, was then stratified into two survival probability categories: low (score 0–4) and high (score 5–7). Table 3 shows the distribution of outcomes across these two risk groups. Among patients classified in the high-probability survival group, 68.57% survived, compared to only 15.3% in the low-probability survival group. The cut-off score > 4 achieved a sensitivity of 81.54%, specificity of 63.87%, positive predictive value (PPV) of 15.30%, and positive likelihood ratio (LR^+^) of 0.66.

**Figure 2:**
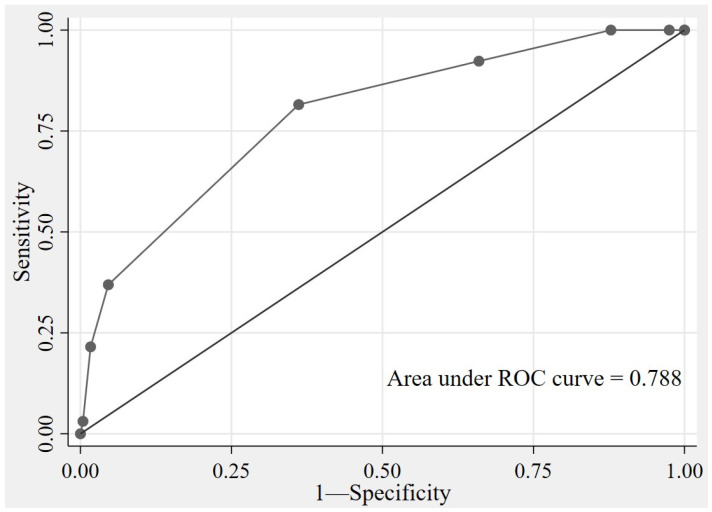
Receiver Operating Characteristic Curve for the Final Model. (AuROC = 0.788, 95% CI: 0.73–0.85).

**Figure 3:**
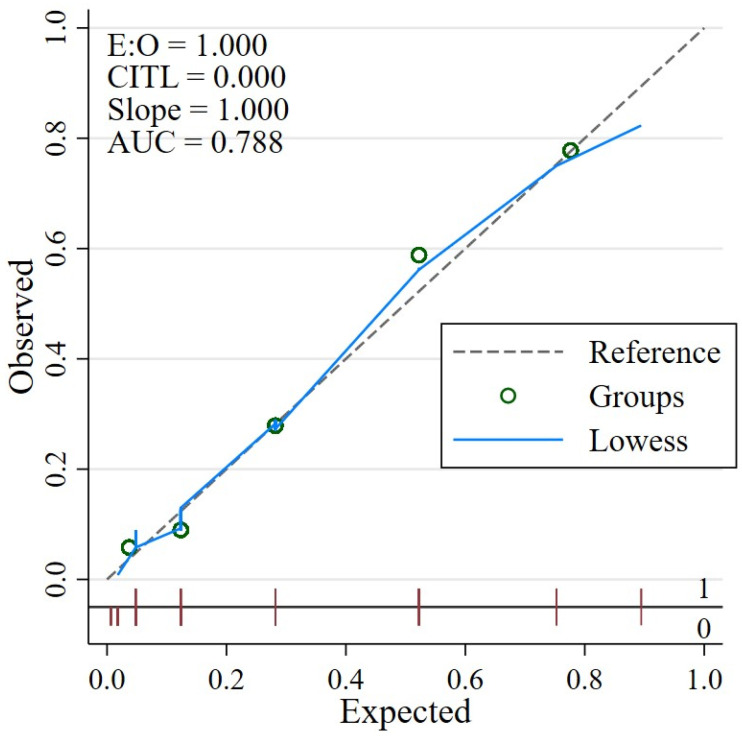
Calibration Plot Comparing Predicted and Observed Survival Probabilities. LOWESS-smoothed curve approximates the diagonal reference line. Hosmer–Lemeshow test: *p* = 0.535.

The model’s discriminative ability, as assessed by the AuROC, was found to be highly effective. With an AuROC of 0.788 (95% CI: 0.73–0.85) the model demonstrated a strong discriminatory performance (Figure 2). The model’s calibration was evaluated using the Hosmer–Lemeshow goodness-of-fit test (*p* = 0.535), and the calibration plot (Figure 3) further confirmed the model’s effectiveness by showing a close agreement between predicted and observed survival probabilities. Internal validation was performed using bootstrap resampling with 1000 replications. The apparent area under the AuROC was 0.826. The optimism estimated through bootstrapping was 0.009, resulting in an optimism-adjusted AuROC of 0.817. This minimal decrease reassures the readers of the model’s good internal validity and low risk of overfitting.

## 4. Discussion

This study presents the POTEC (Platelet count, Oxygen saturation, Time of CPR, Elective surgery, and initial ETCO_2_) Score as a practical clinical tool for predicting 24-h survival following perioperative CPR. The score is based on five routinely available variables: a platelet count of ≥100 × 10^9^/L, preoperative SpO_2_ above 90% on room air, initial ETCO_2_ levels between 35 and 45 mmHg after endotracheal intubation, the requirement for elective surgery, and a CPR duration of 30 min or less. The POTEC Score showed acceptable discrimination (AuROC = 0.788, 95%CI: 0.73–0.85) and demonstrated good calibration, which aligns with established thresholds for clinical prediction models. These results expand upon existing knowledge and address a significant gap in the literature. Most current tools, such as the Cardiac Arrest Survival Post-Resuscitation In-Hospital (CASPRI) and the Good Outcome Following Attempted Resuscitation (GO-FAR), were designed for in-hospital cardiac arrests, either in general wards or ICU settings. They often depend on long-term outcomes or variables that are not readily available in real-time during surgical procedures. In contrast, the POTEC score targets the immediate perioperative period, providing anesthesiologists with a timely and actionable framework for early prognostication and critical decision-making during or shortly after resuscitation [3,4].

This model synthesizes both established and under-recognized predictors into a single, context-specific scoring system. It aligns with prior evidence underscoring the prognostic value of physiologic indicators such as SpO_2_ [16,25] and ETCO_2_ [1,2,26], yet distinguishes itself by integrating these with surgical context variables. Among these, CPR duration emerged as the strongest independent predictor of survival (OR = 11.26, 95% CI: 3.57–35.47), corroborating prior literature linking prolonged resuscitation to poor outcomes [16,23,27,28]. Notably, platelet count is a variable seldom emphasized in previous CPR studies, and it showed significant predictive value. This finding extends existing research suggesting that Thrombocytopenia may reflect underlying perioperative coagulopathy, massive bleeding, or systemic inflammatory states, all of which are highly relevant in surgical settings. Therefore, platelet count in the POTEC score may serve as a proxy marker of both hemostatic reserve and systemic stress, adding a hematologic dimension not typically captured in existing resuscitation models [26,29].

Unlike generic early warning systems such as the MEWS [15,16] or CART Score [14], which lack specificity in operative environments, the POTEC score is grounded in real-time, perioperative physiology. A cutoff score of 4 or higher can assist in making decisions about ICU admissions, prompt the early initiation of advanced hemodynamic monitoring, and aid in prioritizing limited intensive care resources. In contrast, patients with lower scores may be managed with standard monitoring, which helps optimize the allocation of perioperative critical care resources. In contrast to retrospective models that depend on baseline comorbidities or neurologic status (e.g., CASPRI and GO-FAR scores include long-term outcome variables like comorbidities, age, initial arrest rhythm, and neurological baseline [11,27,30,31]. The POTEC score was designed for real-time bedside use during anesthetic emergencies, emphasizing immediacy and simplicity.

Some variables, though statistically significant in univariable analysis, were excluded from multivariable analysis during model derivation due to collinearity or diminished added value. For example, ASA classification and age were associated with survival but offered limited incremental information beyond SpO_2_ and elective status. Initial cardiac rhythm and stability of hemodynamics were similarly of no significance in the multivariable model. These exclusions reflect a refined perspective suggesting that in the perioperative setting. Physiological markers may offer more timely prognostic information than broader comorbidity indices. Interestingly, age ≥ 65 years was associated with better survival; this may reflect trauma-related mortality among younger patients, underscoring the complexity of interpreting age-based outcomes in heterogeneous surgical populations.

Importantly, the intended application of the POTEC score is immediately after ROSC, not as a preoperative screening tool nor for long-term prognosis. The focus on 24-h survival may seem limited; however, this time frame captures the most critical period for resource allocation, escalation of care, and shared decision-making regarding resuscitation outcomes. Short-term survival serves as a pivotal clinical inflection point where real-time prognostication can influence both treatment intensity and communication with patients’ families.

### 4.1. Limitations

Despite the model’s strengths, this study has limitations. It was conducted at a single tertiary-care center in Thailand, which may limit generalizability. The retrospective nature of the data introduces potential bias, particularly in the documentation of CPR quality, ETCO_2_ levels, and exact timing. While internal validation via bootstrapping showed low optimism (0.009), external validation in different settings and populations remains necessary. Although survival for 24h is an important clinical milestone for triage and escalation of care, it does not always predict long-term neurological recovery or functional independence. Future studies should build on this work by investigating how short-term survival affects long-term neurological outcomes, quality of life, and rehabilitation potential.

### 4.2. Implications of All Available Evidence

The POTEC Score makes a meaningful contribution to the field of perioperative medicine by providing a real-time, physiology-based tool for predicting survival after intraoperative cardiac arrest. Its utility lies in prevention through early recognition and tailored triage rather than retrospective evaluation. Identifying patients who are likely to survive the initial 24-h post-arrest period, enables more judicious deployment of intensive resources and avoids potentially non-beneficial interventions. Future directions should explore the integration of this approach into digital anesthesia records, prospective validation across diverse surgical populations, and the potential combination with long-term prognostic indices. In high-stakes perioperative settings, where time is critical and decisions must be made swiftly, the POTEC Score represents a significant step forward in personalized, data-informed resuscitation strategies.

## 5. Conclusions

This study developed and internally validated the POTEC Score, a five-variable tool designed to predict 24-h survival after perioperative cardiac arrest. Unlike existing models, the POTEC Score incorporates real-time, perioperative-specific parameters, offering strong discriminatory performance and calibration. By identifying patients with favorable short-term prognoses, this tool has the potential to inform triage, guide resuscitative efforts, and support resource allocation in critical surgical settings. Future studies should externally validate this model in diverse clinical environments and assess its utility in improving perioperative outcomes through data-driven clinical judgment.

## Figures and Tables

**Table 1:** Clinical Characteristics of Survivor and Non-survivor Groups, Univariable Logistic regression odds ratio, evidence of difference (*p*-value), area under the receiver operating curve with 95% confidence interval. (*n* = 321).

Parameters	*n* Missing	Survivor N (%)	Non-Survivor N (%)	OR (95% CI)	*p*-Value	AuROC (95% CI)
**Age, mean ± SD (years)**	0	60.05 ± 19.57	53.41 ± 20.25			
** Age ≥ 65 years**		32 (49.23)	74 (28.91)	2.58 (1.39–4.79)	0.003	0.602 (0.53–0.67)
**Female**	0	33 (50.77)	81 (31.64)	2.23 (1.28–3.87)	0.005	0.596 (0.53–0.66)
**ASA 1–2**	0	19 (29.23)	7 (2.73)	14.69 (5.84–36.92)	<0.001	0.633 (0.58–0.69)
**Elective surgery**	0	16 (24.62)	17 (6.64)	4.59 (2.17–9.71)	<0.001	0.589 (0.53–0.64)
**Preoperative Spontaneous breathing**	0	23 (35.38)	53 (21.09)	2.05 (1.14–3.69)	0.017	0.572 (0.51–0.64)
**Preoperative no sign of shock**	0	53 (81.54)	146 (57.03)	3.33 (1.69–6.52)	<0.001	0.623 (0.57–0.68)
**Hemoglobin (g/dL), mean ± SD**	3	10.57 ± 2.70	8.87 ± 3.43			
**≥8**		57 (87.69)	152 (60.08)	4.73 (2.16–10.34)	<0.001	0.638 (0.59–0.69)
**Platelet count (10^9^/L)**, **median [Q1, Q3]**	3	202 [162, 291]	138 [87, 217]			
**≥ 100 × 10^9^/L**		58 (89.23)	171 (67.59)	3.97 (1.74–9.08)	0.001	0.608 (0.56–0.65)
**Non-trauma surgery**	0	43 (66.15)	113 (44.14)	2.47 (1.39–4.37)	0.02	0.610 (0.54–0.67)
**Site of operation** 0						
**Upper intra**—**abdominal**		22 (33.85)	100 (39.06)	1.25 (0.71–2.21)	0.440	0.526 (0.46–0.59)
**Major vascular**		9 (13.85)	43 (16.80)	1.26 (0.58–2.73)	0.565	0.515 (0.47–0.56)
**Intracranial**		10 (15.38)	56 (21.88)	1.54 (0.74–3.22)	0.250	0.533 (0.48–0.58)
**Intrathoracic**		12 (18.46)	44 (17.19)	0.92 (0.45–1.85)	0.809	0.506 (0.46–0.52)
**Orthopedic**		2 (3.08)	9 (3.52)	1.15 (0.24–5.45)	0.862	0.502 (0.48–0.53)
**Others**		13 (20.00)	18 (7.03)	3.31 (1.52–7.17)	0.002	0.565 (0.54–0.63)
**Supine position**	0	54 (84.38)	236 (92.16)	0.46 (0.20–1.03)	0.060	0.453 (0.40–0.50)
**Preoperative SpO_2_**, **median, [Q1, Q3]**	14	98 [95, 100]	95.5 [82, 100]			
**≥90%**		55 (84.62)	145 (59.92)	3.68 (1.79–7.57)	<0.001	0.624 (0.57–0.68)
**The initial ETCO_2_ (mmHg),**	14					
**mean ± SD**		28.09 ± 8.62	23.17 ± 10.53			
**<35**		49 (75.38)	214 (88.43)			
**35–45**		16 (24.62)	25 (10.33)	ref		
**>45**		0	3 (1.24)	2.79 (1.38–5.63)	0.004	0.571 (0.52–0.63)
**Time of POCA:** **Working hours**	0	31 (47.69)	153 (59.77)	1.63 (0.94–2.82)	0.08	0.560 (0.49–0.63)
**Initial rhythm before POCA**	0					
**Shockable**		14 (21.54)	35 (13.67)	1.73 (0.87–3.46)	0.118	0.539 (0.48–0.59)
**Non shockable**		51 (78.46)	221 (86.33)			
**Duration of CPR (min),** **Median [Q1, Q3]**	0	10 [5, 20]	20 [10, 35]			
**≤30**		61 (93.85)	180 (70.31)	6.44 (2.26–18.34)	<0.001	0.618 (0.58–0.66)

Values are presented as mean ± standard deviation (SD), median [Q1, Q3], or number (percentage), unless otherwise indicated. Abbreviations: POCA, perioperative cardiac arrest; CPR, cardiopulmonary resuscitation; OR, odds ratio; CI, confidence interval; AuROC, area under the receiver operating characteristic curve; ASA, American Society of Anesthesiologists physical status classification; ETCO_2_, end-tidal carbon dioxide; SpO_2_, peripheral oxygen saturation; g/dL, grams per deciliter; ×10^9^/L, 10^9^ cells per liter; mmHg, millimeters of mercury; min, minutes; SD, standard deviation; Q1, first quartile; Q3, third quartile.

**Table 2:** Item Scoring POTEC Score for Prediction Survive Derivation Using Multivariable Logistic Regression Coefficients.

Potential Predictors	ORs	95% CI	*p*-Value	Coefficients	Score
**P** = Platelet count 100 × 10^9^/L	4.24	1.72–10.41	0.002	1.44	1
**O** = Oxygen saturation ≥ 90% upon OR arrival (SpO_2_)	2.83	1.31–6.14	0.008	1.04	1
**T** = Duration of CPR ≤ 30 min	11.26	3.57–35.47	<0.001	2.42	2
**E** = Elective surgery	4.92	1.97–12.32	0.001	1.59	2
**C** = ETCO_2_ level 35–45 mmHg after intubation	2.99	1.33–6.70	0.008	1.09	1

Abbreviations: ORs, adjusted odds ratios; CI, confidence interval; SpO_2_, peripheral oxygen saturation; ETCO_2_, end-tidal carbon dioxide; CPR, cardiopulmonary resuscitation; ROSC, return of spontaneous circulation; mmHg, millimeters of mercury; ×10^9^/L, 10^9^ cells per liter. Definitions: Platelet count ≥ 100 × 10^9^/L refers to a preoperative platelet concentration above the specified threshold. Oxygen saturation ≥ 90% indicates a peripheral oxygen saturation (SpO_2_) level measured upon arrival in the operating room. Duration of CPR ≤ 30 min is defined as the elapsed time from cardiac arrest to either ROSC or cessation of resuscitative efforts. Elective surgery denotes procedures that are planned, as opposed to emergency operations. ETCO_2_ 35–45 mmHg represents the initial end-tidal carbon dioxide value recorded immediately after endotracheal intubation.

**Table 3:** Distribution of Survival and Death Across Different Probability Categories (Low, and High).

Probability Categories	Total	Low Probability N (%) (score 0–4)	High Probability N (%) (score 5–7)
**Total**	303		
**Survived**	65	41 (15.30)	24 (68.57)
**Died**	238	227 (84.70)	11 (31.43)
**Prognosis performance**			
**Sensitivity**		81.54	36.92
**Specificity**		63.87	95.38
**PPV**		15.30	68.57
**LR^+^**		0.66	7.99
**LR^−^**		0.103	0.741
**95% CI**		0.23–0.40	1.94–3.53

Values are expressed as percentages (%), unless otherwise indicated. Abbreviations: PPV. positive predictive value; LR^+^, positive likelihood ratio; LR^−^, negative likelihood ratio; 95% CI, 95% confidence interval.

## Data Availability

Datasets generated or analyzed in this study are accessible upon reasonable request from the corresponding author.

## Abbreviations

POCA	Perioperative Cardiac Arrest
CPR	Perioperative cardiopulmonary resuscitation
ROSC	The return of spontaneous circulation
TRIPOD	The Transparent Reporting of a Multivariable Prediction Model for Individual Prognosis or Diagnosis guidelines
GA	General anesthesia
RA	Regional anesthesia
MAC	Monitored anesthesia care
ASA	American Society of Anesthesiologists physical status
MAP	Mean arterial pressure
SpO_2_	Peripheral oxygen saturation
ETCO_2_	End-tidal carbon dioxide
AuROC	The receiver operating characteristic curve
SD	Standard deviation
IQR	Interquartile range
ORs	Adjusted odds ratios
CIs	Confidence intervals
CASPRI	The cardiac arrest survival post-resuscitation in-hospital
GO-FAR	The good outcome following attempted resuscitation
CART	The cardiac arrest risk triage score
MEWS	The modified early warning score
